# Supplementary figures and images for: DNA methylome signatures as epigenetic biomarkers of hexanal associated with lung toxicity
Source: PeerJ. 2021 Feb 4;9:e10779. doi: 10.7717/peerj.10779 (PMC7868067; doi:10.7717/peerj.10779)

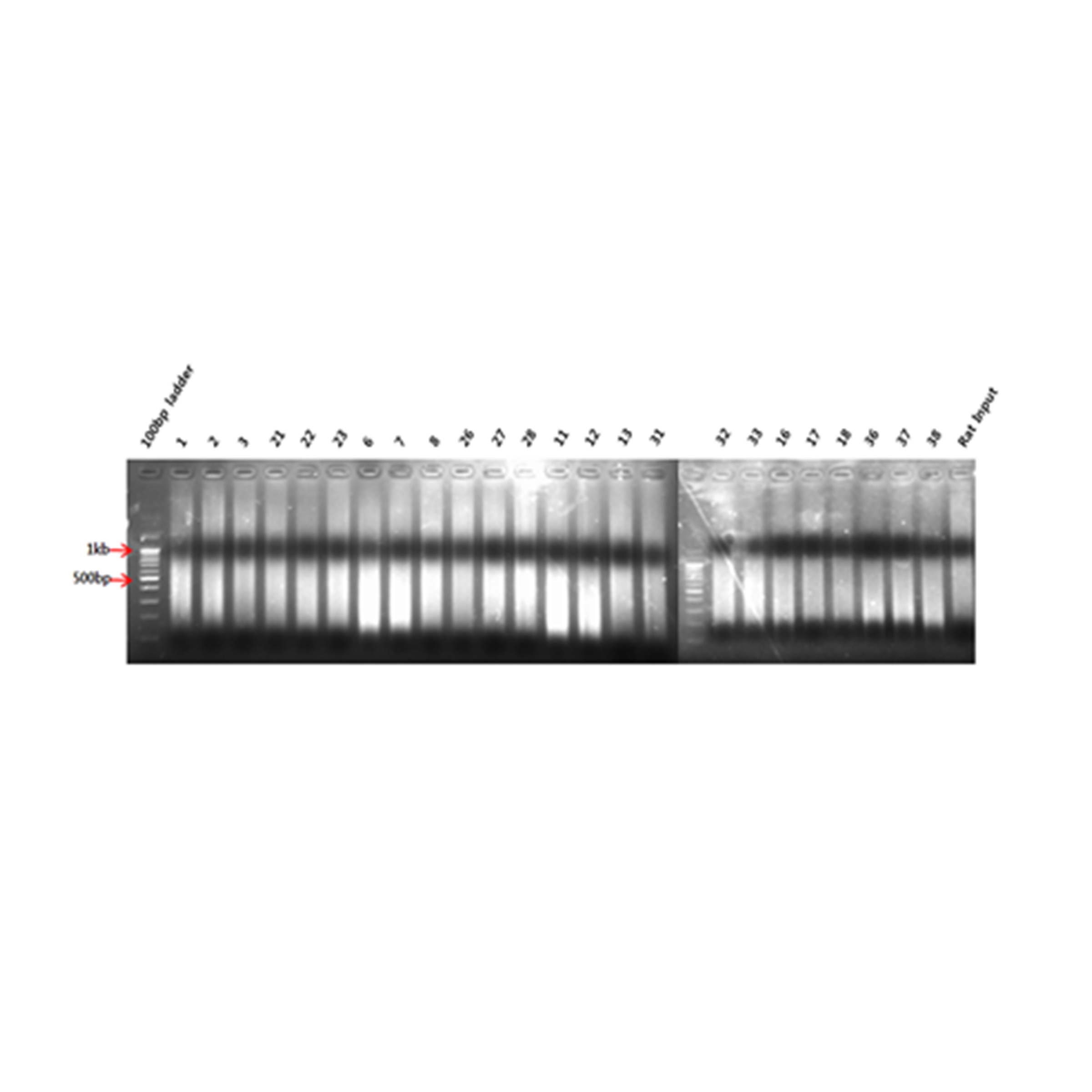

Supplement: Figure S2 [file peerj-09-10779-s001.png]

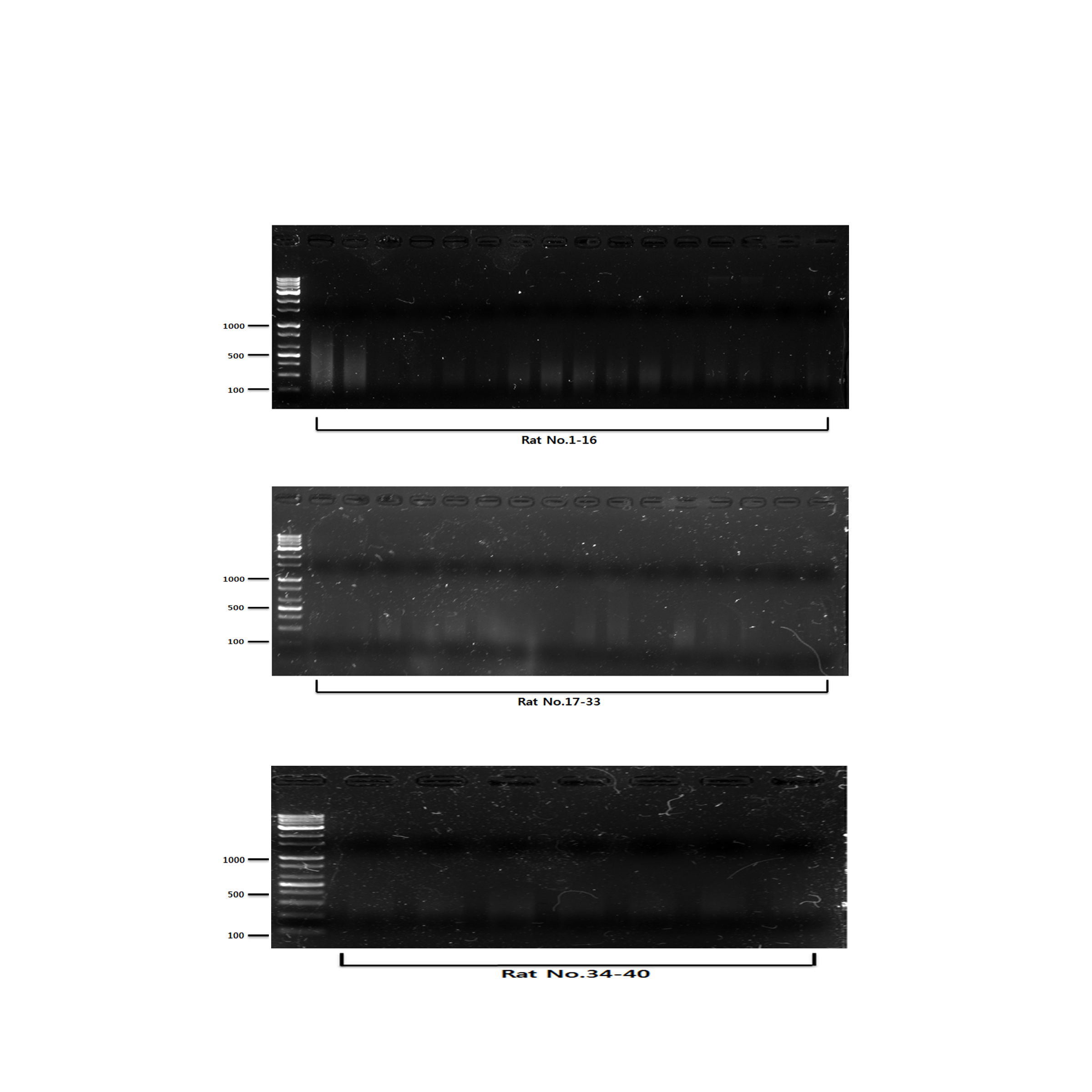

Supplement: Figure S1 [file peerj-09-10779-s003.png]
