# Supplementary material for: DNA methylome signatures as epigenetic biomarkers of hexanal associated with lung toxicity
Source: PeerJ. 2021 Feb 4;9:e10779. doi: 10.7717/peerj.10779 (PMC7868067; doi:10.7717/peerj.10779)
Supplement: Supplemental Information 1 [file peerj-09-10779-s002.docx]

**Miame Checklist**

DNA Methylome Signatures as Epigenetic Biomarkers of Hexanal Associated with Lung Toxicity

Yoon Cho, Mi-Kyung Song, Jae-Chun Ryu

**Part 1 Experiment description**

- **mouse type :** Fischer-344 rat
- **experimental variables (runners vs. non-runners, high fat vs. low fat):** Filtered clean air vs. Hexanal
- **n-count:** n=6/group (Control, low-dose, middle-dose, and high-dose group; a total of 24 DNA samples)
- **tissues used for slide:** Lung tissues from Fischer-344 rats
- **mouse age, and other variables :** 7-8 weeks

**Part 2 Array design.**

- **Array series :** Agilent-066316 Rat CpG 2X400K
- **Deconvoluted spot list with gene names :** https://www.agilent.com/store/productDetail.jsp?catalogId=G4124A
- **Array type (mouse, human, cDNA, oligo, number of genes):** Rat
- **Array size:** 2X400K
- **Slide type (and coating):** Single glass slide formatted with two SurePrint G3 400K arrays

**Part 3 Samples**

- **Cy3/Cy5 labels for tissues:** Yes
- **Dye swap? Or reference control?** None
- **Labelling protocol used:** The methylated DNA sample was labeled with Cy5-dUTP and the input DNA sample was labeled with Cy3-dUTP and 50 μl of master mix mix(dNTPs-dATP, dGTP, dCTP; 120uM, dTTP; 60uM, Cy5-dUTP or Cy3-dUTP; 60uM).
- **Sample extraction protocol used:** Genomic DNA was extracted from the homogenized lung tissue of rats using DNA Mini kit (Qiagen, Germany) following manufacturer's instructions
- **Amount of sample labelled:** 24 samples

**Part 4 Hybridizations**

- **Hybridization protocol:** After purification, the labeled DNA and methylated IP samples were mixed with blocking and hybridization buffer, followed by denaturation for 3 min at 95°C and incubated for 30 min at 37°C. Centrifuge at 17,900 x g for 1 min and immediately placed in a Customized Rat Methylation Microarray (400K) chip and hybridized for 40 h at 65°C in hybridization oven (Agilent Technology, USA).
- **ALL modifications and deviations from the protocol:** None
- **Manual hybridization or automatic chamber?** : Manual hybridization
- **Number of slides done at the same time:** 2 slides
- **Hyb time:** 40 hours
- **Number of washes:** The hybridized microarrays were washed as the manufacturer’s washing protocol (Agilent Technologies, USA).
- **Amount of labelled sample hybridized :** > 5ug
- **Labelling efficiency:** Cy3 labeled incorporation > 3.5 pmole/ul, Cy5 labeled incorporation > 2.5 pmole/ul

**Part 5 Measurements**

- **Which version of scanner software used:** Agilent DNA microarray scanner (Agilent Technologies, USA) and Agilent Feature Extraction software (v10.7.3.1).
- **Laser power for scan:** Agilent DNA microarray scanner (Agilent Technologies, USA)
- **Instrument model numbers:** Agilent SureScan Microarray Scanner G4900DA
- **Must save original .tiff format images (composite image is optional):** Raw data are available on the NCBI Gene Expression Omnibus (GEO) under accession number GSE129313).
- **Normalization protocol:** The all data were normalized using Agilent's Workbench v7.0 Software and the expression pattern of methylation was analyzed in each genomic probe.
- **Does the scanner software subtract background? How much**?: Intensity dependent normalization is a technique that is used to eliminate dye-related artifacts in two-color experiments
- **Spot raw values, background intensity, ch1 and 2 intensity, etc.:** Raw data are available on the NCBI Gene Expression Omnibus (GEO) under accession number GSE129313).
- **Corresponding gene name:** The data are available on the NCBI Gene Expression Omnibus (GEO) under accession number GSE129313).
- **Methods of analysis (MAN, Spotfire, Genespring) be detailed.:** All data were normalized using Agilent's Workbench v7.0 and the expression pattern of methylation was analyzed in each genomic probe. The data were normalized by dividing the average of the signal intensity of the exposed group by the normalized average of the control group. The differentially methylated probes were selected using the 3.0-fold change cutoff.
- **Normalized to controls? Controls removed? All normalization parameters:** All data were normalized using Agilent's Workbench v7.0 and the expression pattern of methylation was analyzed in each genomic probe. The data were normalized by dividing the average of the signal intensity of the exposed group by the normalized average of the control group.
- **Name of Images, Experiment, and location of files.:** Raw data are available on the NCBI Gene Expression Omnibus (GEO) under accession number GSE129313).
- **Lowess or other normalization if used (and parameters):** Normalization was made using the intra-array (Intensity dependent) LOWESS normalization method.
- **Output file:** None
- **Normalized ratios:** None
- **Numerical manipulations:** None
- **Cut off values:** The differentially methylated probes were selected using the 3.0-fold change cutoff.

**Part 6 Normalization controls**

- **Hypothesis:** The normalization process attempts to correct for artifacts caused by non-linear rates of dye incorporation as well as inconsistencies in the relative fluorescence intensity between some red and green dyes.
- **Gene expression patterns found:** Compared with the control group, all three hexanal-exposed groups (Low dose, 600 ppm; Middle dose, 1,000 ppm; High dose, 1,500 ppm) showed distinctly different methylation patterns (Fig.1). The data is the averaged signal that is acquired from normalizing the signal intensity by dividing the average of the signal intensity of the control group.
- **Controls used, normalization methods used (see above):** We used the intra-array (Intensity dependent) LOWESS normalization method. Intensity dependent normalization is a technique that is used to eliminate dye-related artifacts in two-color experiments that cause the cy5/cy3 ratio to be affected by the total intensity of the spot. This normalization process attempts to correct for artifacts caused by non-linear rates of dye incorporation as well as inconsistencies in the relative fluorescence intensity between some red and green dyes.
